# Diphyllobothriasis Associated with Eating Raw Pacific Salmon

**DOI:** 10.3201/eid1506.090132

**Published:** 2009-06

**Authors:** Naoki Arizono, Minoru Yamada, Fukumi Nakamura-Uchiyama, Kenji Ohnishi

**Affiliations:** Kyoto Prefectural University of Medicine, Kyoto, Japan (N. Arizono, M. Yamada); Tokyo Metropolitan Bokutoh Hospital, Tokyo, Japan (F. Nakamura-Uhciyama, K. Ohnishi)

**Keywords:** Tapeworm, diphyllobothriasis, Diphyllobothrium nihonkaiense, salmon, Oncorhynchus, parasites, zoonoses, synopsis

## Abstract

The incidence of human infection with the broad tapeworm *Diphyllobothrium nihonkaiense* has been increasing in urban areas of Japan and in European countries. *D. nihonkaiense* is morphologically similar to but genetically distinct from *D. latum* and exploits anadromous wild Pacific salmon as its second intermediate host. Clinical signs in humans include diarrhea and discharge of the strobila, which can be as long as 12 m. The natural life history and the geographic range of the tapeworm remain to be elucidated, but recent studies have indicated that the brown bear in the northern territories of the Pacific coast region is its natural final host. A recent surge of clinical cases highlights a change in the epidemiologic trend of this tapeworm disease from one of rural populations to a disease of urban populations worldwide who eat seafood as part of a healthy diet.

## Abstract 2

This tapeworm disease is changing from one of rural populations to one of urban populations worldwide.

Broad tapeworms such as *Diphyllobothrium latum* and *D. nihonkaiense* are exotic parasites that grow as long as 12 meters in the small intestine. By the mid-19th century, infection with the Japanese broad tapeworm was known to be contracted by eating salmon (Figure 1) and was considered to be infection with *D. latum* until 1986, when Yamane et al. revised the identification of the Japanese broad tapeworm and established the new species *D. nihonkaiense* (*1*). Both tapeworms exploit freshwater copepods as their first intermediate host. However, in contrast to *D. latum,* which uses freshwater fish such as perch, char, and pike as the second intermediate host, *D. nihonkaiense* uses anadromous fish, *Oncorhynchus* spp., such as *O. masou* (masu salmon), *O. gorbuscha* (pink salmon), and *O. keta* (chum salmon), which migrate across the northern Pacific Ocean to the Sea of Okhotsk and the Bering Sea (*2*,*3*). Recent studies have demonstrated complete mitochondrial genomes of *D. nihonkaiense* and *D. latum* (*4*,*5*). These genomes have not only rendered species diagnosis more reliable, but they have also provided a wealth of genetic markers that could be useful for investigating their population genetics, ecology, and epidemiology.

**Figure 1:**
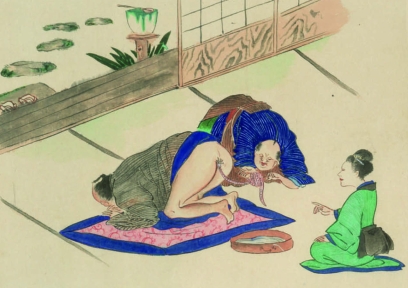
Wood print depicting a man passing a strobila of a broad tapeworm. The caption (not shown) said, “The man ate masu salmon. After a time, a strange object emerged from the anus and was pulled out: it turned out to be 2–3 m long.” From Shinsen Yamaino Soushi, by Daizennosuke Koan (1850). Courtesy of the Tohoku University Medical Library.

Diphyllobothriasis nihonkaiense was once endemic to coastal provinces of central and northern Japan, where salmon fisheries thrived. However, in the past several decades, regions with endemic diphyllobothriasis nihonkaiense have disappeared from Japan, yet the infection has been perpetuated among urban people who eat sushi and sashimi. Although the number of clinical cases of the infection in large cities has fluctuated some in the past 20 years, the incidence was particularly high in 2008. Moreover, clinical cases caused by *D. nihonkaiense* have been emerging even in European countries (*6*–*9*), suggesting that the globalization of this tapeworm disease is probably due to the worldwide expansion of commercial sales of fresh or frozen wild Pacific salmon. We outline the current situation of diphyllobothriasis nihonkaiense in Japan, together with its still-mysterious ecology and life cycle.

## Recent Surge of Pacific Salmon–associated Diphyllobothriasis

We retrospectively examined annual case numbers of diphyllobothriasis nihonkaiense in 2 institutes; the Department of Medical Zoology of the Kyoto Prefectural University of Medicine in Kyoto (MZ) and the Department of Infectious Diseases of the Tokyo Metropolitan Bokutoh Hospital (BH) in Tokyo. MZ is the sole institute specializing in research and diagnosis of parasitic diseases in Kyoto city (population 1.4 million). BH is one of the major public hospitals in metropolitan Tokyo.

From 1988 through 2008, a total of 149 cases of diphyllobothriasis have been recorded: 95 at MZ and 54 at BH. Diphyllobothriasis nihonkaiense was diagnosed by morphologic appearance and taxonomic characteristics of the strobila (body of the mature tapeworm) passed in feces of a person who had a history of eating salmon or a habit of eating sushi or sashimi, which are normally composed of sea fish, often salmon. DNA sequences of the tapeworm *cox*1 and/or *nad*3 genes were also analyzed from most (42) patient specimens obtained since 2004; results confirmed the identification of *D. nihonkaiense*. Molecularly confirmed *D. latum,* from humans or fish, has not been reported in Japan.

**Figure 2:**
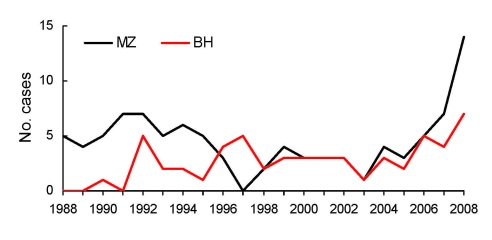
Diphyllobothriasis cases, Department of Medical Zoology of the Kyoto Prefectural University of Medicine in Kyoto and Department of Infectious Diseases of the Tokyo Metropolitan Bokutoh Hospital in Tokyo, Japan, 1988–2008.

Annual incidence rates of the clinical cases at MZ and BH show an apparent surge in recent years (Figure 2). In a broad assumption that the case numbers at MZ represent all cases of this tapeworm infection in Kyoto, the average incidence in the past 20 years was 0.32 cases per 100,000 population per year, and that in 2008 was 1.0 case per 100,000 population. Incidence throughout Japan has not been estimated because a nationwide investigation has never been conducted. Nevertheless, these case numbers at MZ and BH suggest that *D. nihonkaiense* infection is equally as prevalent in Japan as *D. latum is* in some European countries (*10*).

**Figure 3:**
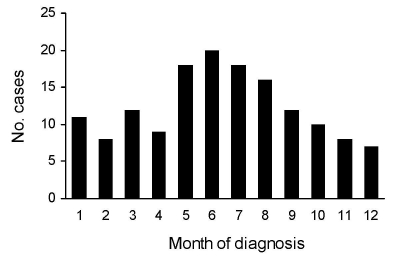
Seasonal occurrence of diphyllobothriasis nihonkaiense, 149 cases, Department of Medical Zoology of the Kyoto Prefectural University of Medicine in Kyoto and Department of Infectious Diseases of the Tokyo Metropolitan Bokutoh Hospital in Tokyo, Japan, 1988–2008.

**Figure 4:**
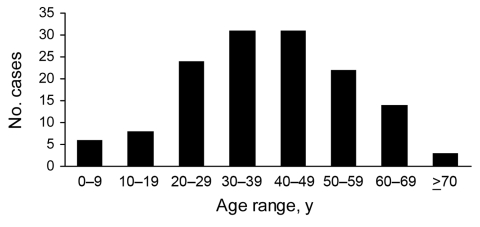
Age distribution of patients with diphyllobothriasis nihonkaiense, Department of Medical Zoology of the Kyoto Prefectural University of Medicine in Kyoto and Department of Infectious Diseases of the Tokyo Metropolitan Bokutoh Hospital in Tokyo, Japan, 1988–2008.

Most patients regularly ate sushi and sashimi. Approximately half could recall that they ate raw or undercooked salmon in the past 6 months. Analyses of 149 cases at MZ and BH showed that the disease occurred during all seasons but that prevalence peaked in early summer (Figure 3). Every age group was affected, from 3 to 77 years. Most patients were 20–59 years of age, which probably reflects more frequent consumption of sushi and sashimi by persons in this age group than in other age groups (Figure 4). Twice as many men than women were affected.

## Signs and Symptoms of Infection

The signs and symptoms caused by *D. nihonkaiense* differ little from those caused by *D. latum*. All 149 patients had consulted physicians after passing tapeworm strobila. Average length of the strobila was 83 cm (range 5–400); patients reported that the strobila tore somewhere along its length when they tried to pull it out. The patients also frequently reported abdominal pain or discomfort and several episodes of diarrhea before passing the strobila, but few complained of substantial weight loss. Of the 149 patients, 73 were treated at MZ, BH, or affiliated institutions. Treatment with anthelminthics (praziquantel for most; bithionol, paromomycin, or sodium amidotrizoate and meglumine amidotrizoate [Gastrografin; Bayer Schering Pharma AG, Berlin, Germany] for a few with older cases) showed that 69 (95%) of 73 patients were infected with 1 tapeworm, 2 were infected with 2 tapeworms, and 2 were infected with 3 tapeworms. The tapeworms obtained measured 50–1,200 cm (average 334 cm). The length of the strobila was not associated with the age or sex of the patient, suggesting that all age groups and both sexes are equally susceptible to this tapeworm.

Pernicious (megaloblastic) anemia has been reported in some patients infected with *D. latum* (*11*). Among the patients with *D. nihonkaiense* infection reported here, low hemoglobin concentration (<12 g/dL) was found in 2 of 43 patients examined. Mild eosinophilia (absolute count >600/μL) was also found in 4 of 37 patients examined. A causal relationship between the anemia or eosinophilia and diphyllobothriasis nihonkaiense for these patients was not determined because neither the type of anemia nor the outcome of anemia or eosinophilia after treatment was examined.

## Wild Pacific Salmon and Risk for Diphyllobothriasis Nihonkaiense

Approximately half of the wild Pacific salmon sold in Japan are caught in the coastal areas of northern Japan, and the other half are imported from Far East Russia and the Pacific coast of North America. Salmon-harvesting rivers run through neither Kyoto nor Tokyo. Suzuki et al. (*12*) investigated plerocercoids (infective larvae) in wild Pacific salmon caught in waters off the coast of northern Japan and sent to Tokyo fish markets during March–July, 2000–2002. They showed that plerocercoids were found in 24 (51%) of 47, 10 (12%) of 82, and 5 (19%) of 27 samples of chum, masu, and pink salmon, respectively. Using PCR-based DNA sequence analysis targeting the *cox*1 and *nad*3 genes, they also showed that all plerocercoids recovered were identified as *D. nihonkaiense* and that 26 chum salmon caught during autumn lacked such infection. This finding implies that wild salmon caught in spring and early summer pose a higher risk for human infection than autumn-caught salmon, consistent with the observation that the incidence of human infection peaks in early summer (Figure 3).

Whether all salmon harvested in the coastal waters off Japan originated from rivers in Japan is unknown. Oshima and Wakai (*13*) investigated the characteristics of masu salmon harboring diphyllobothriid plerocercoids; rate of infection was 27%. They suggested that these masu salmon probably originated from rivers in Russia despite having been captured in the waters off the coast of Japan and unloaded at Japanese ports. To the contrary, an investigation of mature masu salmon captured in the rivers in Hokkaido showed a plerocercoid infection rate of 20%, although no plerocercoids were found in masu salmon juveniles that stayed in the rivers for 1.5 years before migrating to the sea (*14*).

Thus, although earlier exhaustive studies have indicated that the first intermediate host of *D. nihonkaiense* is the freshwater zooplanktonic copepod *Cyclops strenuus* (*15*), whether freshwater is the place of transmission of the parasite from the copepod to salmon remains controversial. Some researchers have been examining a hypothesis that Japanese masu salmon are infected with the plerocercoid not in freshwater but in the sea during their migration through the Sea of Okhotsk, possibly through another intermediate host that links the freshwater copepod and the wild salmon at sea (*14*). So far, no such intermediate host has been discovered.

## Geographic Distribution of *D. nihonkaiense*

Until recently, diphyllobothriasis nihonkaiense had been reported almost exclusively in Japan. In northern communities bordering the Pacific, several additional diphyllobothriid species—*D. klebanovskii*, *D. ursi*, *D. latum*, *D. dendriticum*, and *D. dalliae*—have been implicated in human infections (*16*–*20*). In Far East Russia, *D. klebanovskii,* which also uses wild Pacific salmon as its second intermediate host, is the most common cause of human diphyllobothriasis (*16*,*17*). Recent molecular studies of the DNA sequences of the 18S rDNA, internal transcribed region 1, *cox*1, and *nad*3, clearly indicated the synonymy of *D. klebanovskii* to *D. nihonkaiense*, indicating that *D. nihonkaiense* is distributed not only in Japan but also in Far East Russia up to the Kamchatka Peninsula and that brown bears are its natural final host (*21*).

**Figure 5:**
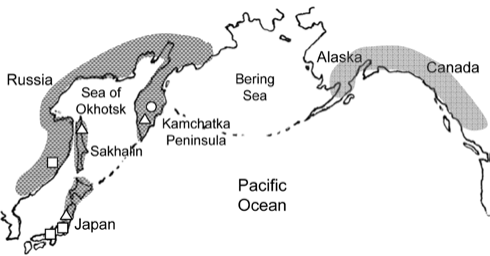
Possible distribution area of *Diphyllobothrium nihonkaiense*. Open circle, open square, and open triangle represent brown bears, humans, and Pacific salmon, respectively, from which *D. nihonkaiense* adult worms or plerocercoids were isolated and identified by DNA sequencing (DNA sequences refer to reference *21*). Patients in European countries are suspected to have eaten salmon imported from the Pacific coast of North America.

In 1980, on the Pacific coast of the United States, an outbreak of diphyllobothriasis was associated with consumption of Pacific salmon (*22*), but species identification of the tapeworm was not conducted. More recently, several clinical cases diagnosed by tapeworm DNA sequencing as *D. nihonkaiense* have emerged in Europe (*6*–*8*). These patients had eaten raw Pacific salmon, probably imported from the Pacific coast of North America. Another case, in a tourist to North America who had eaten raw sockeye salmon from British Columbia, was also diagnosed as caused by *D. nihonkaiense*. (*9*). These reports suggest a far broader geographic distribution *of D. nihonkaiense* than previously believed (Figure 5).

However, whether *D. nihonkaiense* in these regions consists of a biologically homogeneous population is still uncertain. The most enigmatic result of the molecular studies of *D. nihonkaiense* *cox*1 and *nad*3 genes is the presence of 2 deeply divergent lineages that are not defined by the localities of the samples examined so far (*21*). Thus, further studies are needed to look for an association between the host species and/or geographic localities and the 2 genotypes of *D. nihonkaiense*.

## Other Diphyllobothriid Tapeworms in Salmon

*D. nihonkaiense* is not the sole tapeworm species carried by wild Pacific salmon. On the Pacific coast of North America, *D. ursi* has been isolated from brown bears, black bears, and humans (*18*,*19*,*23*,*24*). The plerocercoid of *D. ursi* is found predominantly in sockeye salmon (*O. nerka*) and occasionally in coho salmon (*O. kisutch*)*.* A major difference between *D. ursi* and *D. nihonkaiense* (*D. klebanovskii*) is their plerocercoid stage: plerocercoids of *D. ursi* encyst on stomach serosa of salmon (*18*), and plerocercoids of *D. nihonkaiense* (*D. klebanovskii*) have been found mainly in the body musculature of chum, masu, and pink salmon (*1*–*3*). In some South American countries, cultivated Atlantic salmon (*Salmo salar*) have been implicated as the source of *D. latum* infection (*25*,*26*).

## Conclusions

The epidemiology of diphyllobothriasis nihonkaiense has changed drastically from rural to urban areas because of the rapid expansion of the transport system for fresh and frozen fish to meet a demand for seafood in healthy diets. The uninterrupted occurrence of diphyllobothriasis nihonkaiense in urban areas implies that the *D. nihonkaiense* tapeworm perpetuates its natural life cycle successfully between salmon and its final host animals in northern territories of the Pacific Ocean; however, its definite natural life cycle remains to be elucidated. Freezing and storing at –20 °C for 7 days or –35 °C until solid and storing at –35 °C for 15 hours is sufficient to kill parasites, although these conditions may not be suitable for freezing particularly large fish, e.g., those thicker than 6 inches (*27*).

It seems that the general public in Japan is only vaguely aware of the possible risk for parasitic diseases associated with eating sushi and sashimi made from marine fish. Although some information on this health risk is provided through means such as health education programs open to the public or television programs, the emphasis is generally on the risk for anisakiasis, one of the most prevalent parasitic diseases among Japanese. Persons are generally underinformed, especially about the risk of diphyllobothriasis from eating raw salmon. Moreover, people like sushi and sashimi made of never-frozen fish far better than that made from frozen fish. Consumers and retailers should be made aware of the risk for tapeworm infection posed by eating raw or undercooked wild salmon.
